# Cropping System Conversion led to Organic Carbon Change in China’s Mollisols Regions

**DOI:** 10.1038/s41598-017-18270-5

**Published:** 2017-12-22

**Authors:** Yuxin Tong, Jianguo Liu, Xiaolin Li, Jing Sun, Anna Herzberger, Dan Wei, Weifeng Zhang, Zhengxia Dou, Fusuo Zhang

**Affiliations:** 1 Key Laboratory of Plant-Soil Interactions, Ministry of Education, Center for Resources, Environment, and Food Security, China Agricultural University, Beijing, 100193 China; 2 Key Laboratory of Soil Environment and Plant Nutrition of Heilongjiang Province, Fertilizer Engineering Technology Research Center of Heilongjiang Province, Institute of Soil Fertilizer and Environment Resources, Heilongjiang Academy of Agriculture Sciences, Harbin, 150086 China; 3 Center for Systems Integration and Sustainability, Department of Fisheries and Wildlife, Michigan State University, East Lansing, MI 48824 USA; 4 Center for Animal Health and Productivity, School of Veterinary Medicine, University of Pennsylvania, Kennett Square, PA 19348 USA

## Abstract

Land use change driven by diet, globalization, and technology advancement have greatly influenced agricultural production and environment in the mollisols region of China, with a marked impact on the depletion of soil organic matter, a signature property of mollisols. Here we report findings on soil organic carbon (SOC) change in three different cropping systems (soybean, soybean/maize, corn) in Northeast China during a 10-year time span. The results indicated that the decline rate of SOC in recent ten years (0.27 g kg^−1^ yr^−1^) has slowed down considerably compared to previous decades (1.12 g kg^−1^ yr^−1^). Crop system conversion from soybean monocropping to corn monocropping or break system was the critical factor for SOC change, and the background SOC was the second influence factor. When approaching a SOC turning point, conversion from low carbon input crop system (soybeans monocropping) to high carbon input crop system helped slow down the SOC decline (break crop) or even improve SOC (corn monocropping) in mollisols regions. This result implied that imported soybean has brought benefit for Northeast China. But for sustainable goal in China’s mollisols region, straw returning, optimized nitrogen fertilization and no tillage are all necessary whatever in continues maize or rotation system.

## Introduction

Mollisols (black soils) are highly productive soils that cover 4.23 million km^2^ in the world, accounting for 3.2% terrestrial surface and 28.6% farmland among all soil types. The crop output of mollisols in USA corn belt, Northeast China and Ukraine has been increasing dramatically and dominate food production enhancement in global scale during the past two decades, owing to the high soil fertility and intensive farming practices^1,2^. However, mollisols regions are facing the challenge of soil degradation, caused by erosion, acidification, and decreasing soil organic matter^3,4^. Higher soil organic carbon (SOC) content is a fundamental property. However, it is easily lost when agriculture was introduced. For example, average SOC of mollisols in Northeast China has been 100 g kg^−1^ in nature situation, but it could be decreased by half after land reclamation, and even continually decline if with inappropriate farming management^5,6^. The declining SOC not only have negatively impact on soil productivity, but also became an important factor for climate change due to greenhouse gases emission^7^. Thus, maintain or improving soil fertility of mollisols is important for sustainability of global agriculture.

Northeast China is one of three main mollisols regions in the world, with a total of 70 thousand km^2^ ^2,8^. This area has become an important food basket since 1950s due to the fertile soil, producing 15.9%, 33.6%, and 33.9% of rice, corn, and soybeans of whole China in 2014^9^. However, the growth rate of crop yield has decreased considerably since 2000, approaching a nearly steady phase despite continued increase in fertilizer use as well as expansion in irrigated area^9^. Of various obstacles occurring in this region, declining SOC is most noteworthy. From 1950s to 1980s, SOC decreased by more than half (from 103 to 43.2 g kg^−1^), caused by the combined effects of land reclamation, declined aggregate stability, and decreased porosity^10^. In the following two decades (from 1980s to 2000s), the decreasing SOC continued and reached an averaged value of 20.8 g kg^−1^ in 2000, mainly due to low-level agricultural production practices and limited biogenic material input^11^. But the magnitude of SOC changes and the driving forces in recent ten years has not been reported.

Theoretically, SOC change is determined by initial SOC stocks, tillage practices, nitrogen fertilization, and carbon input^5,12^. In high initial SOC area, especially in mollisols, the SOC decline could be slow down if proper management practices were adopted. And the SOC could even be contained or recovery when it reach the turning point^5^. The results from long term field experiments (soybeans and corn) in China and US showed that the turning point of mollisols is between 5 to 20 g kg^−1^ depends on management practices^5,13,14^. In the end of last century (2000), SOC in China’s mollisols regions was already very close to this turning point (20.8 g kg^−1^)^11^. Therefore, it is critical time to distinguish key driving forces and take proper counter-measures in order to maintain or improve SOC in China’s mollisols regions.

Tillage management is an important factor for SOC change, but its effect was significant affected by residue retained and cropping systems^15^. N fertilization is another key factor that can influence biomass production and alter SOC storage patterns^16,17^. Usually N fertilization is various greatly among different crop systems, i.e higher input for maize vs. lower input for soybean. Carbon input associated with biomass production, crop rotation intensity and straw return was reported as another key factor^18^. Different cropping systems have different biomass production, and then bring different quantities and qualities of C into the soil and affect soil carbon emission and conservation. Clearly, crop system conversion could affect tillage, nitrogen and carbons input, and finally have influence on SOC change. However, the mechanism has not been well discussed in previous studies, especially in large scale farmland^4,19,20^.

In last ten years, the Northeast China has been experiencing significant changes on farming practices associated with cropping system conversion. Soybeans, a dominant crop in this region adapted to soil and climate conditions, has been largely replaced by corn since 2005 because of outcompeted by imported soybeans from Brazil and the USA^21,22^, especially after the adoption of early-maturity corn varieties and big machinery for mechanization. The statistic data show that soybean planting area declined from 4.96 to 3.07 million ha during 2005–2014 in Northeast China, and the area for corn increased from 7.2 to 14.2 million ha^9^. The cropping system conversion was caused by socioeconomic changes^23^, but its long-term environmental impacts, especially on SOC has not been well evaluated.

In this study, we hypothesize that the conversion of cropping systems could be a new driving force for SOC changes in mollisols of Northeast China in recent decades. Our primary goal is to determine the SOC in farmer’s fields resulting from the conversion of cropping systems and to elucidate the relevant driving factors. Specific objectives are: (1) measure SOC change in corn, soybeans, and corn-soybean break crop systems in mollisols of Northeast China; (2) distinguish key factors that drive the SOC change in different crop systems; (3) project possible measures to sustain or improve SOC.

## Results

### Soil organic carbon change

**Figure 1:**
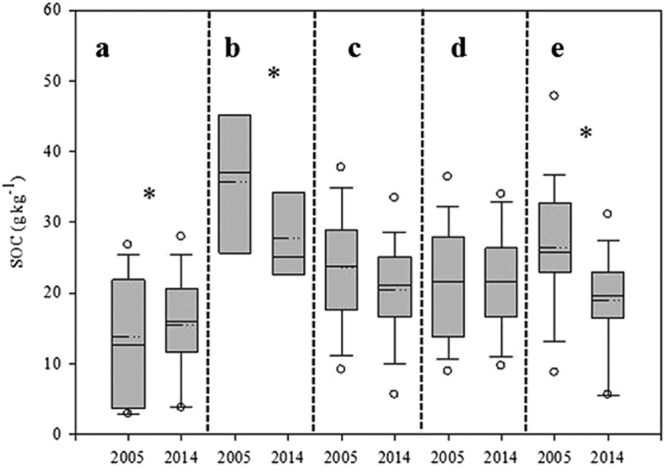
Soil organic carbon changes from 2005 to 2014 in response to different cropping systems. (**a**) Corn monocropping (C-C, *n* = 20), (**b**) soybeans monocropping (S-S, *n* = 5), (**c**) break crop (S-C, *n* = 55). (**d**) Corn/soybean ≥1 (S-C_1_, *n* = 34). (**e**) Corn/soybean from 0 to 1 (S-C_2_, *n* = 21). Between each cropping system, means followed by asterisk are significantly different (*P* < 0.05).

Over all 80 soils/sampling sites, averaged SOC decreased from 21.9 g kg^−1^ in 2005 to 19.2 g kg^−1^ in 2014 (or by 12.3%, *P* < 0.05). The variation of SOC among the samples decreased from ±10.3 g kg^−1^ in 2005 to ±7.2 g kg^−1^ in 2014, which was ascribing to the uniform management practice in past ten years. SOC in C-C system (corn monocropping) increased from 13.8 to 15.5 g kg^−1^ (+12.3%) (Fig. 1a), but decreased from 35.8 to 22.2 g kg^−1^ (−38.0%) in the S-S (soybean monocropping) and from 23.5 to 20.3 g kg^−1^ (−13.6%) in the S-C system (break system) (Fig. 1b and c). In S-C group, when the ratio of corn/soybean greater or equal to 1, no significant different SOC changes found between 2005 and 2014 (Fig. 1d). When the ratio of corn/soybean less than 1, the SOC significantly decreased from 26.3 g kg^−1^ in 2005 to 19.0 g kg^−1^ in 2014 (Fig. 1e). These results demonstrate that in farmer’s field, different cropping systems has led to substantial different SOC change.

Accordingly, calculated SOC stock in the top 0–20 cm soil profile increased by 12.7% (from 36.2 Mg ha^−1^ to 40.8 Mg ha^−1^) for the C-C system, and decreased by 37.7% (35.8 Mg ha^−1^ to 22.3 Mg ha^−1^) and 9.6% (55.9 Mg ha^−1^ to 50.5 Mg ha^−1^) for S-S and S-C system, respectively. The changes of SOC stock in different crop system were in consistent with the trend of SOC change because BD did not change in each crop system between 2005 and 2014 (although BD was significantly different among all cropping systems) (Table 1).

**Table 1:** Comparison of SOC stock change and BD change between different cropping system in both 2005 and 2014.

	Cropping systems			Initial SOC	
	C-C	S-S	S-C	HISOC	LISOC
Samples	20	5	55	49	31
BD in 2005 (g cm^−3^)	1.37 ± 0.08 a	1.12 ± 0.09 b	1.23 ± 0.11 b	1.17 ± 0.10 B	1.40 ± 0.04A
BD in 2014 (g cm^−3^)	1.35 ± 0.06 a	1.27 ± 0.06 a	1.27 ± 0.08 a	1.24 ± 0.07 B	1.38 ± 0.06A
Interannual T test	ns	ns	ns	ns	ns
SOC stock change(Mg kg^−1^)	4.63 ± 8.71 a	−13.5 ± 3.35 c	−5.4 ± 12.8 b	−5.08 ± 7.81 B	1.14 ± 6.85A

HISOC: the samples with SOC ≥20 g kg^−1^ in 2005; LISOC: the samples with SOC < 20 g kg^−1^ in 2005; BD: bulk density; ns: no significant difference. Data are means ± SD. Year treatment indicates the comparison of average BD between 2005 and 2014. Within a line, cropping systems means followed by the same letter are not significantly (*P* < 0.05).

### Driving forces for soil organic carbon change

#### Initial soil organic carbon

**Figure 2:**
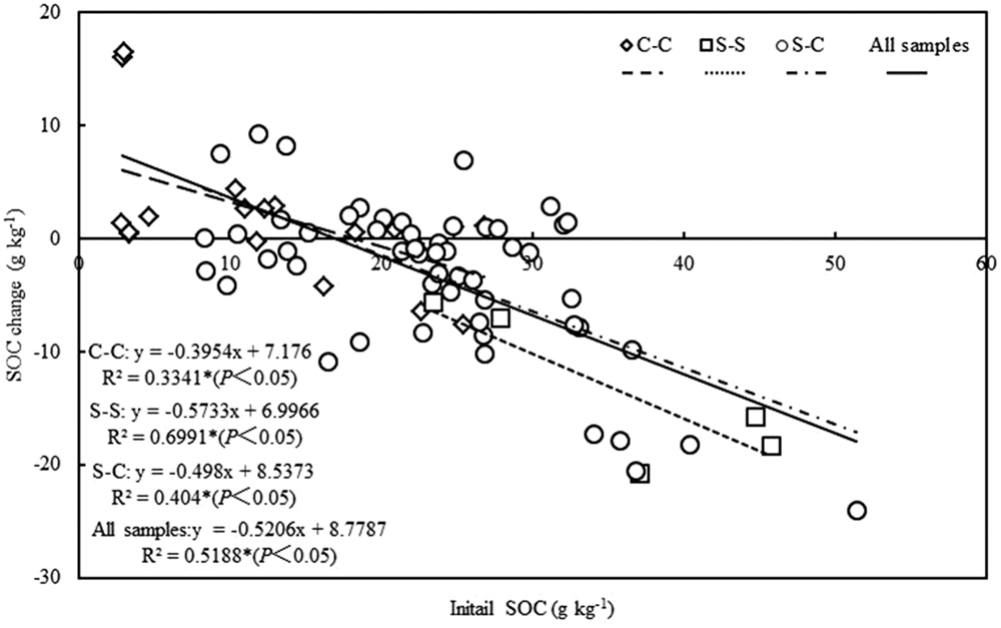
Correlation between SOC change from 2005 to 2014 and initial SOC in 2005. Each point represents the data collected from one farmer field site. Triangle indicated C-C systems (n = 20). Square indicated S-S systems (n = 5). Circle indicated S-C systems (n = 55). Asterisk is significantly different (*P* < 0.05).

**Figure 3:**
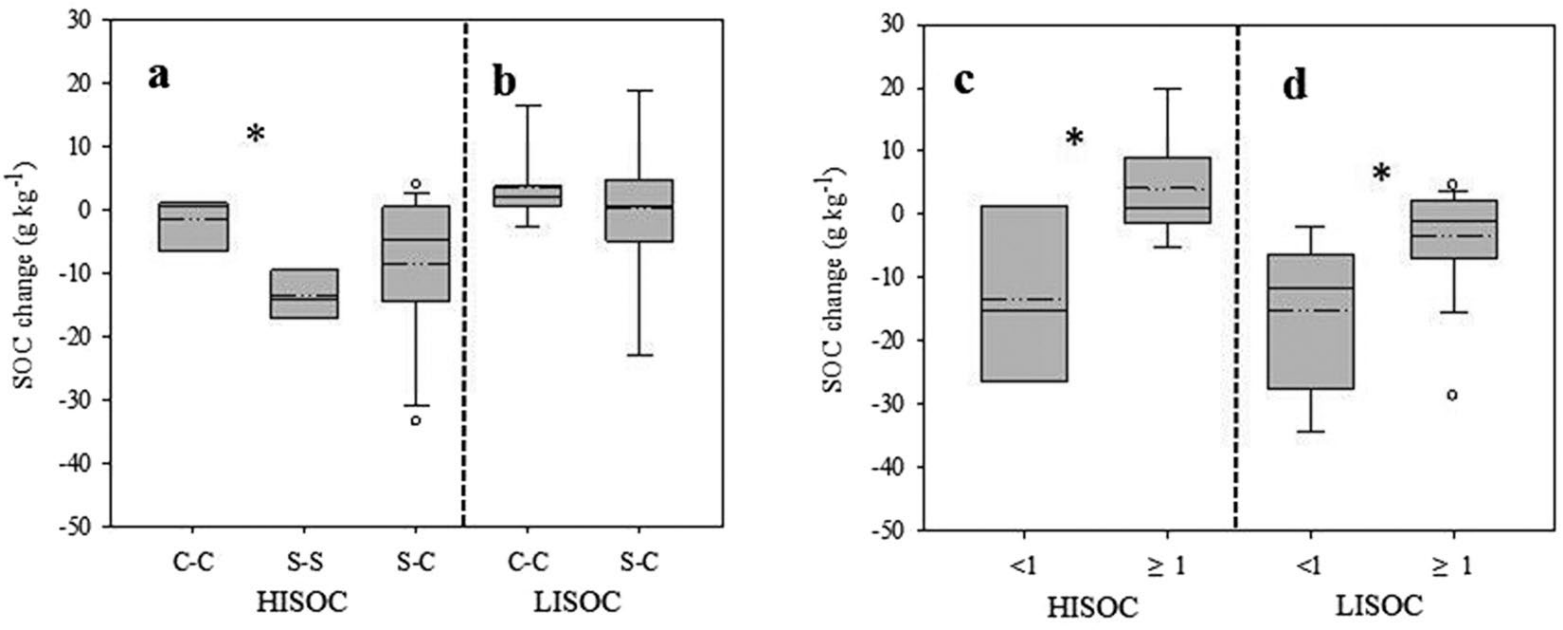
SOC change of cropping systems under different initial SOC. (**a**) SOC change of C-C, S-S, and S-C under HISOC (high initial SOC). (**b**) SOC change of C-C and S-C under LISOC (low initial SOC). (**c**) SOC change of S-C system under HISOC. (**d**) SOC change of S-C system under LISOC. <1 means the duration of corn/soybean in the 10 year time span is less than 1. ≥1 means the duration of corn/soybean in the 10 year time span is equal to or greater than 1. Between each cropping system, means followed by asterisk are significantly different (*P* < 0.05).

Over all 80 sampling sites, SOC changes have a linear relationship with the initial SOC (or background SOC in 2005), termed the higher initial SOC the bigger SOC changes (Fig. 2). Same trends were also found in C-C, S-S and S-C systems. All samples were further divided into two groups according to the initial SOC (HISOC: initial SOC in 2005 ≥20 g kg^−1^, LISOC: initial SOC in 2005 <20 g kg^−1^). Under HISOC, the decreasing rate of SOC in S-S (−13.5 g kg^−1^) was significantly higher than that in C-C (−1.53 g kg^−1^) and S-C (−8.66 g kg^−1^) (*P* < 0.05) (Fig. 3a). Under LISOC, there is no significant different of SOC change between C-C (3.48 g kg^−1^) and S-C (0.21 g kg^−1^) (Fig. 3b).

In S-C system, whatever under HISOC or LISOC, the decreasing rate of SOC in high corn/soybean ratio group was always lower than that in low corn/soybean ration group (Fig. 3c and d). This funding showed an interaction between SOC changes, initial SOC, and cropping systems. The initial SOC determined the direction of SOC changes (i.e go up or go down), however the magnitude of SOC changes depends on the crop system. Regardless of initial organic carbon levels, the more corn grown, the smaller the organic carbon decreased.

When estimated the turning point (the point of initial SOC where SOC changes equal to 0) by using the regression function established for different crop systems in Fig. 2, different value were found for different cropping systems, such as 18.2 g kg^−1^ for C-C, 12.2 g kg^−1^ for S-S and 17.1 g kg^−1^ for S-C. This funding implied that the turning point determined by crop system.

#### Carbon input

According to equation (3), aggregated carbon input from crop roots averaged 0.97, 0.16, and 0.62 Mg ha^−1^ per year for C-C, S-S, and S-C systems, respectively. As for carbon input from straw, farmer survey indicated that 95% of soybean straw and 45% of corn straw were removed from the cropland (detail information show in Fig. S2b). Therefore, carbon input from straw amounted 3.26 Mg ha^−1^ per year for C-C but only 0.05 Mg ha^−1^ per year for S-S and 1.73 Mg ha^−1^ per year for S-C (Table 2). The rhizodeposition carbon inputs were 0.65, 0.1, and 0.42 Mg ha^−1^ per year for the C-C, S-S, and S-C, respectively. Sum up all the sources, carbon input totaled 4.93 Mg ha^−1^ per year for C-C, 0.35 Mg ha^−1^ per year for S-S, and 2.82 Mg ha^−1^ per year for S-C systems, respectively. Carbon input was significant different between cropping systems and straw is demonstrating reason for the gap between cropping systems.

**Table 2:** Annual average carbon input in three cropping systems during 2005 to 2014.

Cropping system	Carbon input (Mg ha^−1^)					
	Straw return	Root	Rhizodeposition	Fertilizer	Seed	Total
**C-C**	3.26 ± 0.42 a	0.97 ± 0.13 a	0.65 ± 0.08 a	0.06 ± 0.02 a	0.01 ± 0.01 a	4.93 ± 0.61 a
**S-S**	0.05 ± 0.01 c	0.16 ± 0.04 c	0.10 ± 0.03 c	0.02 ± 0.01 c	0.02 ± 0.01 ab	0.35 ± 0.09 c
**S-C**	1.73 ± 0.28 b	0.62 ± 0.09 b	0.42 ± 0.06 b	0.04 ± 0.02 ab	0.02 ± 0.01 c	2.82 ± 0.41 b

Data are means ± SD. Within a column, cropping systems means followed by the same letter are not significantly (*P* < 0.05).

#### Nitrogen input

Different cropping system has significant different N input and crop yield, i.e. N input and crop yield for C-C was higher than S-C and S-S (Fig. S2a and c). No significant changing N input and crop yield (fertilizer N plus bio-fixation N) were found in C-C and S-S system during the ten years period. But significant increased N input and crop yield was found in S-C system due to increased corn planting area. When calculated the nitrogen surplus (surplus = N input- N uptake) for each crop system, the surplus is small, and there is no significant difference between monocropping systems and between years, i.e. 10.1, 3.2 kg ha^−1^ for C-C and S-S in 2005, and 15.4, 14.8 kg ha^−1^ in 2014, respectively (Table 3). This result proved that N fertilizer was nearly balanced for soybeans and cron, and not a constraint factors for crop production. According to the farmer survey, the fertilizer products did not change in this region as well as the comprising C content. Therefore, N was not the key factor for carbon input and SOC change in China’s mollisols.

**Table 3:** Nitrogen input, uptake and surplus for different cropping systems in both 2005 and 2014.

Cropping system	N input in 2005 (kg ha^−1^)	N input in 2014 (kg ha^−1^)	Interannual T test	N uptake in 2005 (kg ha^−1^)	N uptake in 2014 (kg ha^−1^)	Interannual T test	N surplus in 2005 (kg ha^−1^)	N surplus in 2014 (kg ha^−1^)
**C-C** ^(a)^	146 ± 19.9	154 ± 20.1	ns	136 ± 16.9	139 ± 22.8	ns	10.1	15.4
**S-S** ^(b)^	178 ± 6.2	180 ± 5.40	ns	175 ± 75.8	165 ± 27.7	ns	3.2	14.8
**S-C** ^(c)^	61.7 ± 39.2	131 ± 31.3	s	57.6 ± 17.6	119 ± 24.3	s	4.1	12.3

N input: N fertilizer + N fixation, N fixation ≈146 kg ha^−1^ ^42^. Data are means ± SD. ns means no significant difference, *P* < 0.05.

#### Factors’ contribution to the soil organic carbon change

Multiple linear regression (MLR) was conducted to identify the driving forces for SOC changes during 2005 to 2014. A significant relationship was established (*p* < 0.05) between SOC changes and the selecting 5 factors (temperature, precipitation, initial SOC, N fertilizer input, carbon input), which explained 73% SOC changes during 2005–2014 (Table 4). VIF (Variance Inflation Factor) and tolerance values of all the explanatory variables were between 0.10 and 10, which means no serious collinearity among all variables.

**Table 4:** Quantification the contribution of all drivers forces on SOC change (*n* = 80).

Influence factors	SOC change		Collinearity Statistics	
	Coefficient	Sig	Tolerance	VIF
Temperature	0.13	0.249	0.29	3.41
Precipitation	−0.04	0.732	0.29	3.47
Initial SOC	−0.27	0.002	0.5	1.99
N fertilizer input	0.03	0.783	0.44	2.29
Carbon input	0.68	<0.001	0.39	2.58
R^2^	0.75			
Adjust R^2^	0.73			
P	<0.001			

VIF, variance inflation factor. Sig, significant different (*P* < 0.05).

Among the 5 factors, Temperature, precipitation and N fertilizer input are not significantly affect the SOC change (*p* < 0.05). However, carbon input and initial SOC have significant effects on SOC change. Carbon input show positive effect but initial SOC show negative effect, which means higher carbon input will increase SOC but higher initial SOC will generate higher decrease of SOC. The interaction between carbon input and initial SOC determine the direction and magnitude of SOC changes.

## Discussion

Estimated SOC in the mollisols region of Northeast China was 103 g kg^−1^ (grassland) in 1950, with a steady decline to 43.2 g kg^−1^ by 1980, 21.9 g kg^−1^ by 2005, and further down to19.2 g kg^−1^ by 2014 in this study^6^. Generally, the decreasing rate was slow down in recent years (from 1.12 g kg^−1^ yr^−1^ during 1980s to 2000s, to 0.27 g kg^−1^ yr^−1^ during 2005 to 2014), which mainly ascribe to the relative lower initial SOC and cropping system conversion in last ten years. The increased SOC observed in most sites implied current SOC could reach the turning point, but was significantly determined by management practices associated with crop system (the estimated turning points are 18.2 g kg^−1^ in C-C, 17.1 g kg^−1^ in S-C and12.2 g kg^−1^ in S-S, Fig. 2). Therefore, it is critical time to adopt right solution to maintain or improve SOC in China’s mollisols regions.

Theoretically, the function of single solution was widely tested in previous researches, such as tillage, nitrogen (N) fertilization, and crop residue management^5^. Inconsistent performances were always found because the interaction of all these factors and specific SOC level were ignored^24–26^. Our result indicated that the effect of same solution is different under different initial SOC. For example, C-C can increase the SOC under LISOC situation but only can slow down the decline rate of SOC in HISOC site (Fig. 3). Unfortunately, most studies on improving SOC were located in low initial SOC site^27^. We argue to conduct multiple factor research targeting specific SOC conditions.

The carbon input model and the multiple factor regression method has successfully explained the contribution of various factors on SOC changes in this study. It is clearly show high biomass crop with high straw return rate is dominate reason for SOC changes whatever under high initial SOC regions or low initial SOC regions. In general, this method could explain 73% reasons for SOC change (Table 4), and the rest 27% mainly because of the ignored carbon output (very limited data in this region). Carbon outputs include heterotrophic respiration, soluble carbon leaching and runoff^28^. Previous studies reported heterotrophic respiration of soybeans (3.56 Mg ha^−1^) to be much greater than that of corn (1.58 Mg ha^−1^) in mollisols region of Northeast China^29,30^, because rhizosphere respiration for N fixation of soybean is high^29^. Meanwhile, carbon loss through leaching and runoff represented about 5% of the net fixed C^31^. Considering these carbon output, the carbon budget gap between corn and soybean would be greater.

This research found break crop system (S-C) did not improve SOC whatever under higher initial SOC sites or lower initial SOC sites. It is different to the results in previous researches at experiment stations in the mollisols region of Northeast China. For example, it was reported that SOC in rotation system (S-C system) increased from 15.5 to 18.83 g kg^−1^ after 28 years, or increased from 17.2 to 21.9 g kg^−1^ after 19 years in mollisols region of northeast China^32,33^. The reason for the difference was caused by the different farming practice and initial SOC. Notably, researches in experiment station were often conducted with the best management practices (manure or straw return) and targeted in lower initial SOC regions. We can imagine, if current rotation or break crop systems in farmer’s field be improved by returning soybean straw, this system could improve SOC.

This result implied corn monocropping system (C-C) may be the best solution to maintain or improve SOC in mollisols regions. However, there may be tradeoffs associated with monocropping of corn, such as pests and diseases, soil erosion, and other environmental issues related to nutrient losses. Relevant researches suggested that rotation or break crop systems are better than monocropping system, especially on rational utilization of resources (such as nitrogen fixation from legume can be used for next season’s crop) and decrease of pests and diseases^34–36^. To improve the management practices in current cropping system (rotation, fertilizer, straw treatment, tillage) to ensure high carbon input and to minimize the side effect should be grand challenge for sustainable development of mollisols regions.

## Conclusions

Our data indicated that SOC decreased by 12.3% in the study area during 2005 to 2014. However, the changes of SOC are significantly different in various cropping systems, i.e. 38.0% decrease in soybean monocropping, 13.6% decrease in break system, while 12.3% increase in corn monocropping. In farmland of mollisols region, initial SOC has been a dominant factor for SOC change, but crop system conversion associated with specific practices is a new factor in recent ten years. Whether in high initial SOC condition or low initial SOC condition, continued soybeans monocropping would not be sustainable in terms of SOC decline. Continued corn monocropping would enhance SOC but other side effect should be addressed. The break cropping system did not increase SOC due to failure in return soybean straw into field. To improve current cropping systems, better rotation, straw return, optimized nitrogen fertilization and tillage are all essential for sustainable development of mollisols regions.

## Materials and Methods

### Site description

Songnen Plain was chosen as study area, which is located in Western Heilongjiang Province, Northeast China. The study area share 54.2% mollisols (38 thousand km^2^) in China and produces approximately 72% soybeans in the molisols region in China^8,9^. Songnen Plain has a relatively short history in agricultural production, with land reclamation from natural grassland to cropland occurring in the 1950s. Characterized by typical continental monsoon climate, annual temperature in the study area was nearly stable during 2005–2014 based on data from four weather stations although annual precipitation varied from 400 to 800 cm^3^ (Fig. S1).

### Sample collection

**Figure 4:**
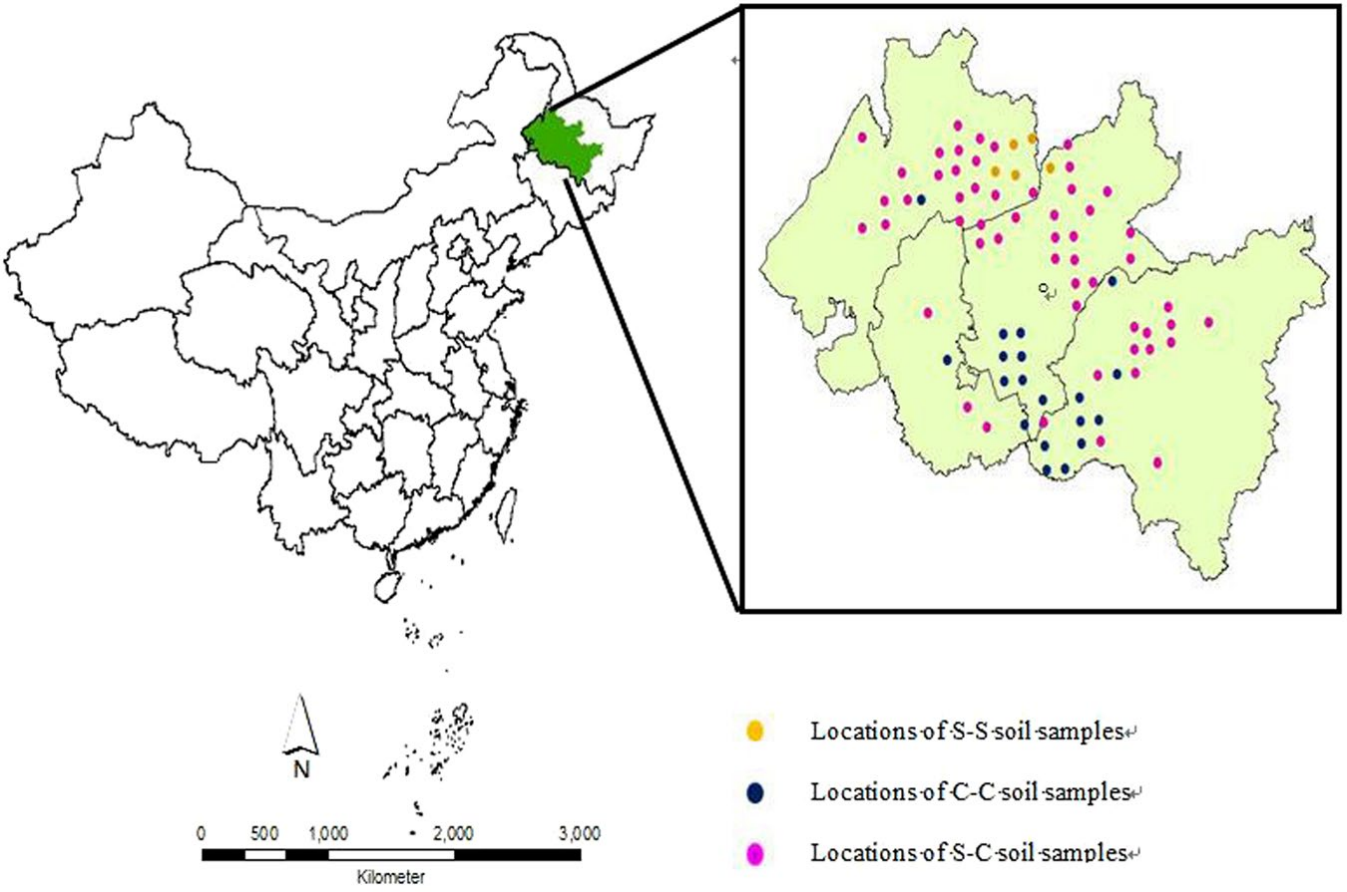
The locations of sampling sites in Heilongjiang Province, China. Blue circles indicate corn monocropping during 2005–2014 (C-C), yellow circles indicate soybean monocropping during 2005–2014 (S-S), pink circles indicate break crop during 2005–2014 (S-C). Samples for C-C, S-S and S-C were 20, 5 and 55 respectively. The map created by the ArcGIS 10.2 software package. http://www.esri.com/software/arcgis.

Main cropping systems in the study area comprised of break system (corn-soybean), corn monocropping, and soybean monocropping from 1970s to 2000s. To identify the SOC change caused by cropping system conversions, we monitored 80 mollisols soil sites in 21 counties of Songnen Plain, and took soil samples for each site in 2005 and 2014 respectively (Fig. 4). The monitoring sites were selected per each 400 km^2^ areas of mollisols region based on GPS (Global Position System). Each soil sample was a composite of five random cores from the 0–20 cm soil profile within 0.5 ha area of crop field which was belonging to an individual farmer. Bulk density (BD) samples were taken at same time. Two bulk density sub-samples were taken in each random core, one from a wheel tracked inter-row and one from an untracked inter-row.

Farmers who host each of the sampling land were surveyed (face to face interview), and their information regarding crops, yield, and management practices (e.g. fertilizer application, tillage, crop straw usage, etc.) for each year (during 2005–2014) were recorded. The surveyed information was used to analyze the driving force of SOC change in each sample sites. Time series data of crop yield, straw return, and nitrogen fertilizer input of each monitoring site showed in Fig. S2. Straw returning rate is defined as percentage of farmers retaining all crop residues in land. The increasing crop yield and N input of S-C was caused by the increasing sites of corn from 2005 to 2014 (Fig. S2). According to the survey, the fertilizer products did not change as well as the comprising C content. We found ridge tillage (20 cm) was adopted for each site on both of corn and soybean in the ten year span.

We divided the 80 samples into three categories according to different cropping systems: corn monocropping (C-C), soybean monocropping (S-S), and break crop (S-C) during the period of 2005 and 2014. Before the study (2005), there were 20 C-C, 32 S-S, and 28 S-C (all sites stay over 5 years until 2005). During 2005 and 2014, the number and locations of C-C remained the same as that in 2005, but number of S-S decreased to 5 sites. There were 27 S-S converted to S-C system and the 20 previous S-C have no Change. Therefore, the S-C system increased to 55 sites. To better understand the crop conversion in S-C system, we divided the 55 sites into two sub categories according to the duration of corn and soybean in the 10 year time span, for example: corn years/soybean years ≥1 (S-C_1_, n = 34), corn years/soybean years <1 (S-C_2_, n = 21) (Fig. S3). To better understand the effect of initial SOC on SOC changes, we divided 80 samples to high initial SOC (HISOC, the samples with SOC ≥ 20 g kg^−1^ in 2005) and low initial SOC (LISOC, the samples with SOC < 20 g kg^−1^ in 2005).

### Estimation of soil organic carbon stocks

Soil organic matter (SOM) was determined by using the potassium dichromate method^37^, then SOC was calculated by using Equation (1). The latter is often used to evaluate SOC change^38,39^.

1 $${\rm{SOC}}=0.58\times {\rm{SOM}}$$

Subsequently, soil organic carbon stock in top soil (0–20 cm) was calculated using Equation (2):

2 $${{\rm{C}}}_{{\rm{s}}}={\rm{SOC}}\times {\rm{BD}}\times 0.2/10$$

Where Cs is soil organic carbon stock (Mg ha^−1^), SOC is soil organic carbon content (g kg^−1^), BD is soil bulk density (g cm^−3^), and 0.2 m is soil thickness (20 cm in this research).

### Estimation of the change in carbon input

To quantify the contribution of changing cropping systems on SOC, the aggregated soil carbon input (SCI) during 2005 and 2014 was calculated as Equation (3)^40^:

3 $${\rm{SCI}}={{\rm{C}}}_{{\rm{straw}}}+{{\rm{C}}}_{{\rm{root}}}+{{\rm{C}}}_{{\rm{seed}}}+{{\rm{C}}}_{{\rm{fertilizer}}}+{{\rm{C}}}_{{\rm{rhizodeposition}}}$$

Where C_straw_ and C_root_ are the amount of carbon returned through root and straw. C_seed_ and C_fertilizer_ are the amounts of carbon added to soil by seed and chemical fertilizer (urea)^40^. According to the farmer survey, nitrogen fertilizer was urea-based compound fertilizer or urea. C_rhizodeposition_ is carbon added to the soil through rhizodeposition.

Annual grain yields in each site were obtained from farmer survey (Fig. S2a). The straw biomass of all samples was estimated by using the relationship between grain and straw, which was determined by local field experiments during 2001 and 2014 (Equations 4 and 5). The management practices and crop yield of these experiments were similar to the survey farmers. Especially the tillage practice was same to the survey (Table. S1).

4 $${{\rm{S}}}_{{\rm{s}}}=1.0818{{\rm{G}}}_{{\rm{s}}}+269.6\quad {{\rm{R}}}^{2}=0.537$$

5 $${{\rm{S}}}_{{\rm{c}}}=0.8509{{\rm{G}}}_{{\rm{c}}}+3974.1\quad {{\rm{R}}}^{{\rm{2}}}=0.582$$

Where S_s_ is the biomass of soybean straw (kg ha^−1^), G_s_ is soybean grain yield (kg ha^−1^), S_c_ is biomass of corn straw (kg ha^−1^), and G_c_ is corn grain yield (kg ha^−1^).

The biomass of roots was estimated by using the ratio of straw to roots, which was cited from local research on several main varieties of corn and soybeans in mollisols region, i.e. 1.26: 0.33 for soybeans and 1.24: 0.28 for corn^41^. The carbon contents in grain, straw, roots, seed and fertilizer are available in the supplementary.

### Quantification the contribution of all drivers forces on SOC change

Multiple linear regression (MLR) was performed to identify the relationships between environmental variables and SOC by using the SPSS software 13.0 (SPSS Inc.). Temperature, precipitation, initial SOC, N input and carbon input were chosen as the independent variables, whereas SOC change was the dependent variables. More details are available in the supplementary.

### Statistical analysis

The differences of each index among various cropping systems were analyzed with the unpaired T test and ANOVA, the differences of each index between 2005 and 2014 were analyzed with the paired T test in SPSS software (*P* < 0.05 considered as “statistically significant”). Microsoft Excel was used for data processing and regression analysis, and it was also used for drawing along with SigmaPlot and ArcGIS 10.2.

## Electronic supplementary material

Supplementary information
