# Supplementary material for: Cropping System Conversion led to Organic Carbon Change in China’s Mollisols Regions
Source: Sci Rep. 2017 Dec 22;7:18064. doi: 10.1038/s41598-017-18270-5 (PMC5741738; doi:10.1038/s41598-017-18270-5)
Supplement: Supplementary file 1 — Supplementary information [file 41598_2017_18270_MOESM1_ESM.doc]

**Cropping System Conversion led to** **Organic Carbon Change in China’s Mollisols Regions**

Yuxin Tong^1,2^, JianguoLiu^3^,Xiaolin Li^1^, Jing Sun^3^, Anna Herzberger^3^, Dan Wei^2^, Weifeng Zhang^1*^, Zhengxia Dou^4^&Fusuo Zhang^1^

^1^Key Laboratory of Plant-Soil Interactions, Ministry of Education, Center for Resources, Environment, and Food Security, China Agricultural University,Beijing 100193, China.

^2^Key Laboratory of Soil Environment and Plant Nutrition of Heilongjiang Province, Fertilizer Engineering Technology Research Center of Heilongjiang Province, Institute of Soil Fertilizer and Environment Resources, Heilongjiang Academy of Agriculture Sciences, Harbin 150086, China.

^3^Center for Systems Integration and Sustainability, Department of Fisheries and Wildlife, Michigan State University, East Lansing, MI 48824, USA.

^4^Center for Animal Health and Productivity, School of Veterinary Medicine, University of Pennsylvania, Kennett Square, PA 19348, USA.

*Corresponding author ([wfzhang@126.com](mailto:wfzhang@126.com)).


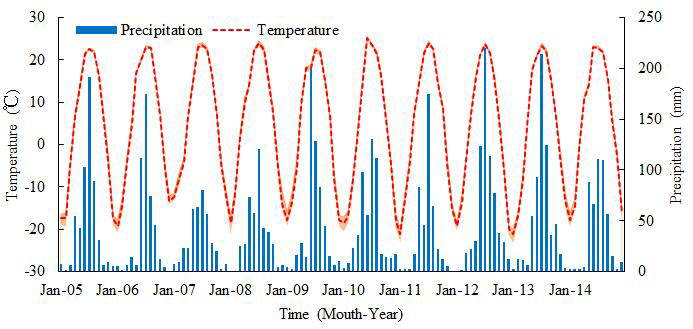


Figure S1. Monthly averaged air temperature and precipitation of 4 weather stations in research regions from 2005 to 2014. Data of air temperature and precipitation are mean of 4 weather stations.


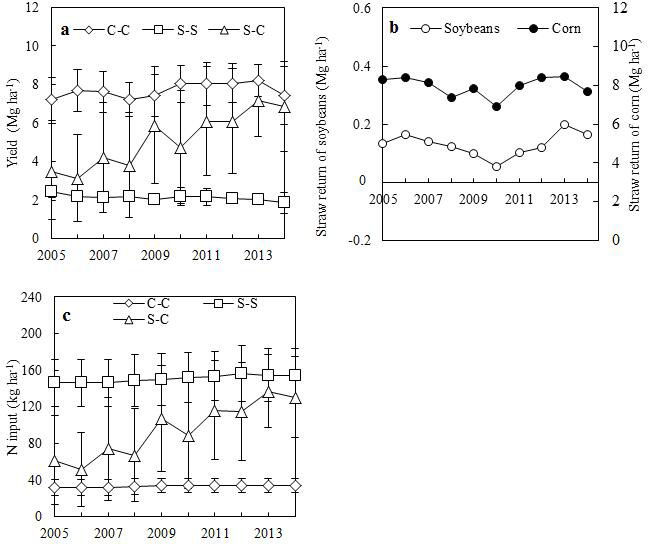


Figure S2.The management practices changes of surveyed crop systems during 2005-2014. (a), average yield, (b), straw returning rate, and (c), N input.


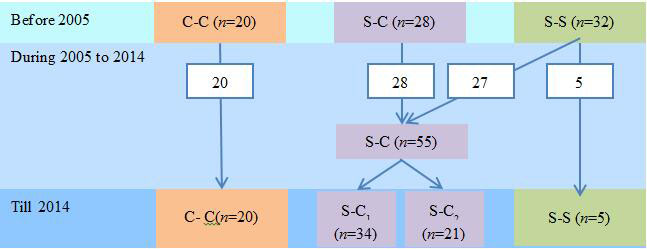


Figure S3. Crop systems conversion of 80 monitored samples before 2005 and during 2005- 2014. C-C, corn monocropping. S-S, soybean monocropping. S-C, break crop. S-C_1_, corn years / soybean years ≥ 1. S-C_2_, corn years / soybean years < 1.

Table S1. Information of experiments used to establish Equations 4 and 5.

| Crop | Sites  (counties) | Numbers | Time  (year) | Tillage type  (method) | Tillage depth  (cm) | Tillage time  (month) | N fertilizer input  (kg ha^-1^) | Yield  (t ha^-1^) |
| --- | --- | --- | --- | --- | --- | --- | --- | --- |
| Soybeans | 10 | 127 | 2003-2009 | ridge | 20 | early May | 30-70 | 1.2-3.7 |
| Corn | 6 | 412 | 2001-2014 | ridge | 20 | early May | 128-210 | 5.1-12.8 |

**Materials and Methods**

**Estimation of the change in carbon input**

The carbon contents in grain, straw, and roots are 43.6%, 44.5%, and 26.2% for soybeans and 45.6%, 45.3%, and 44.8% for corn, respectively . The annual variation of carbon content for each crop was small during last ten years [^3^](#_ENREF_3), then these constant carbon contents coefficients were adopted for annual carbon input estimation. The carbon input from seed and fertilizer was calculated by the C content of grain and urea. The data of seed input and urea application was collected from farmer survey. The rhizodeposition carbon was estimated by using the ratio of rhizodeposition carbon to root biomass carbon: rhizodeposition carbon represented 40% of root biomass carbon in both corn and soybeans [^4^](#_ENREF_4).

**Quantification the contribution of all drivers forces on SOC change**

Multiple linear regression (MLR) was performed to identify the relationships between environmental variables and SOC by using the SPSS software 13.0 (SPSS Inc.). This model was established by Lindsay R, and was applied to evaluate SOC changes in mollisols region [^5^](#_ENREF_5). In these researches, precipitation, temperature, erosion, cropland, forest, fertilizer and grain yield were included. But in this research, according to the main influences factors of SOC in cropland, temperature, precipitation, initial SOC, N input and carbon input were chosen as the independent variables, whereas SOC change was the dependent variables. Due to uniform practices on tillage in all crop system across the research time span, which was excluded in the MLR analysis.

Y=
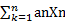
 = a0 + a1X1 + a2X2 + a3X3 + … + anXn (1)

Where Y is the SOC change (g kg^-1^), Xn（n=1，2，3….n）are influence factors, a_n_（n=1，2，3….n） are regression coefficients, a_0_ is regression residuals.

Many reports have indicated that inter-correlation between one independent variable and other independent variables can seriously decreased the accuracy of the regression model [^5^](#_ENREF_5). It is generally assumed that a tolerance of less than 0.10 indicates serious collinearity, a Variance Inflation Factor (VIF) of more than 10 is an indication of severe collinearity[^6^](#_ENREF_6).

1 Miao, H. T., Zhang, W. J., Huang, S. M. & Xu, M. G. Effects of long-term fertilization on assimilated carbon content and distribution proportion of maize in fluvio-aquic soil. *Scientia Agricultura Sinica*. **43**, 4852-4861 (2010).

2 Liang, Y., Han, X. Z., Qiao, Y. F., Li, L. J. & You, M. Y. Soil respiration and carbon budget in black soils of wheat maize-soybean rotation system. *Chinese Journal of Eco-Agriculture.* **20**, 395-401 (2012).

3 Zhang, J., Wang, X. J., Wang, J. P. & Wang, W. X. Carbon and nitrogen contents in typical plants and soil profiles in Yanqi Basin of Northwest China. *Journal of Integrative Agriculture*. **13**, 648-656 (2014).

4 Buyanovsky, G., Brown, J. & Wagner, G. Evaluation of Soil Organic Matter Models. 295-300 (Springer, 1996).

5 Lindsay, R. Doing quantitative research in education with SPSS. *British Journal of Educational Technology*. **36**, 353-354 (2005).

6 O’brien, R. M. A caution regarding rules of thumb for variance inflation factors. *Quality & Quantity*. **41**, 673-690 (2007).
